# Three-dimensional fracture mapping and analysis of coronal fractures in AO/OTA types 33-B3 and C3

**DOI:** 10.1186/s13018-022-03327-7

**Published:** 2022-10-01

**Authors:** Yin Ding, Dong Wang, Muhammad Zeeshan Waheed, Jun-Lin Zhou

**Affiliations:** Department of Orthopedics, Beijing Chaoyang Hospital, Capital Medical University, Beijing, 100020 China

**Keywords:** Supracondylar–intercondylar fracture, Coronal fracture, Distal femur, Morphology, Fracture map

## Abstract

**Background:**

Although the relatively high incidence of coronal fractures in the supracondylar–intercondylar fractures is well established, little is currently known about the morphology of those fractures. Herein, we characterized the coronal fractures in AO/OTA type 33-C3 and assessed their differences with Busch–Hoffa fractures (33-B3).

**Methods:**

We retrospectively collected 61 cases of AO/OTA type 33-B or C fractures with coronal plane fragments and generated three-dimensional fracture maps of those with coronal fractures based on CT imaging and measured angle *α* (the angle between the coronal fracture and the posterior condyle axis in the axis plane) and angle *β* (the angle between the coronal fracture and the posterior femoral cortex in the sagittal plane).

**Results:**

Thirty-three cases (32%) of AO/OTA type 33-C fractures contained coronal fragments. Most of them were type 33-C3 fractures. Angles *α* and *β* for type 33-C3 were significantly smaller than for type B3 at the lateral condyle, while the angles at the medial condyle were not significantly different. The fracture maps showed that the coronal fractures and the articular comminution area were more anterior in type 33-C3.

**Conclusions:**

The incidence of coronal fractures was 32% and 67% in AO/OTA types 33-C and 33-C3, respectively. Our findings suggest that coronal fractures differed between both types, emphasizing the potential need for different treatment approaches.

## Introduction

The incidence of distal femoral fracture is approximately 8.7/100,000/year and exhibits an increasing trend as the population ages [1]. In the past, this fracture exhibited a bimodal distribution, primarily affecting young people who sustained high-energy injuries and older adults subject to low-energy injuries. An increasing body of evidence from recently published studies suggests that older women with low-energy injuries are also susceptible [2, 3], and male patients are often younger than females [1, 4]. Arbeitsgemeinschaft für Osteosynthesefragen/Orthopaedic Trauma Association (AO/OTA) type 33-C3 fractures have a comminuted articular surface, coronal fractures complicated 38.1% of type 33-C fractures in the posterior condyle [5].

In the past, an isolated coronal fracture in the posterior condyle was called Busch–Hoffa fracture and classified as AO/OTA type 33-B3-2 or 33-B3-3. This fracture has attracted significant interest since it is sometimes invisible on plain radiographs [6], with a high incidence of secondary displacement without surgical fixation [7], and the entry point for fixations is through the articular surface [8, 9]. Nowadays, the Letenneur [10] and AO/OTA classifications are the most commonly used in clinical practice. Li et al. [11] and Chandrabose et al. [12] defined new classification methods based on computer tomography (CT) scans. Moreover, Xie et al. described the fracture morphology of Busch–Hoffa fractures by three-dimensional (3D) fracture maps [13].

Few studies have addressed the Busch–Hoffa fragment in AO/OTA type 33-C3 fracture. It is widely acknowledged that identifying fracture characteristics is essential during the preoperative workup. Therefore, this article focused on the morphology of the posterior condylar fragment in AO/OTA type 33-C3 and Busch–Hoffa fracture (B3). We sought to assess the incidence, three-dimensional morphology, and angles of coronal fractures in distal femoral fractures.

## Method and patients

We retrospectively analyzed 186 patients with AO/OTA type B or C fractures in Beijing Chaoyang Hospital from 2013 to 2019. The exclusion criteria were: 1. pathological fractures, periprosthetic fractures, or fractures after fixation; 2. readmission, malunion or nonunion; 3. age ≤ 18 years; and 4. missing preoperative CT images. Among the remaining 166 patients, only cases with a coronal fracture (*n* = 59) were included in our study and classified into type C3 (*n* = 31) and type B3 (*n* = 28).

We recorded the age, gender, and side of injury and collected preoperative CT images (stored as DICOM). Three researchers independently verified CT data. Points of disagreement were resolved by a discussion with a senior surgeon. This study was approved by the Ethics Committee of Beijing Chaoyang Hospital.

### Reconstruction of fractures and fracture map

The methods used to reconstruct and draw maps were based on the literature [4, 13]. The CT data were imported into the medical image analysis software suite: Materialise Mimics Medical 21, and the fractures were reconstructed and reduced virtually. After adjusting the size according to a standard template, the data were transferred to module 3-Matic Medical 13.0 of the same software suite, and the reconstructed and reduced fracture fragments were aligned with the template. The fracture lines and comminution zones were drawn with smooth lines. We drew the 3-D fracture maps by overlapping all fracture lines and comminution zones. Heatmaps were generated by E-3D software (http://www.e3d-med.com) and only showed coronal fractures that separated the posterior condyle.

### Angle measurement

**Fig. 1:**
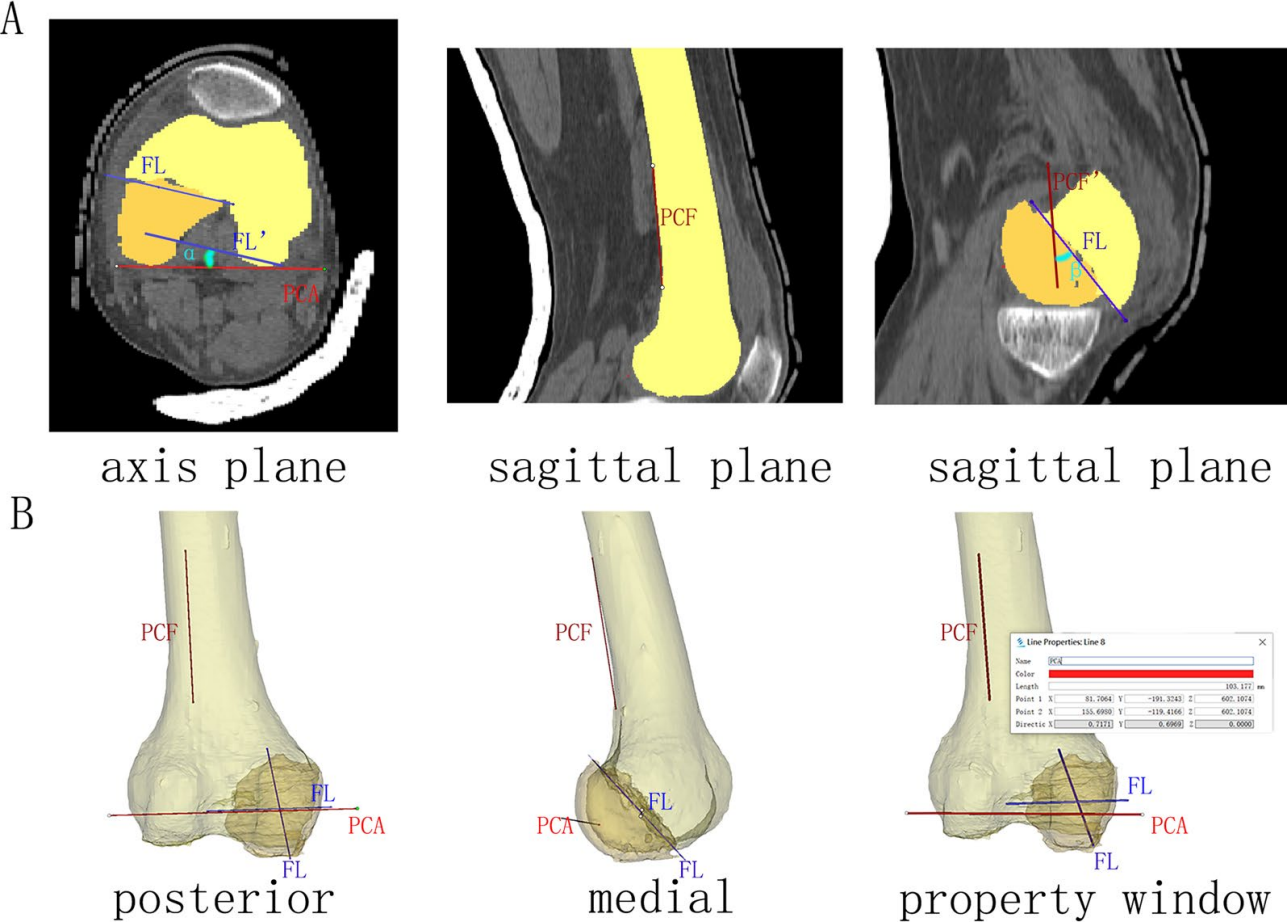
Aimed lines and angles. **A** CT images of the right knee. **B** 3D model of the right knee. The property window shows the vector (directic) of the PCA. PCA, the posterior condylar axis; FL, the fracture line; PCF, the line of the posterior shaft

The angles were measured after reduction. We used the posterior condylar axis (PCA, a line tangent to the most prominent point of both posterior condyles) and the posterior shaft axis (PCF, a line parallel to the posterior cortex of the distal femur) as the reference lines to calculate the fracture angles. Angle *α* was defined as the angle between the coronal fracture line and the PCA in the axial plane, and angle *β* was defined as the angle between the coronal fracture line and PCF in the sagittal plane (Fig. 1).

The angle *α* was positive if the fracture at the lateral condyle extended from an anterolateral to a posteromedial direction or the fracture line at the medial condyle fracture from an anteromedial to posterolateral direction; otherwise, a negative *α* value was recorded. Angle *β* was positive if the fracture line was from an anteroinferior to posterosuperior direction; otherwise, a negative value was recorded.

### Statistics

IBM SPSS Statistics 26 was used. Continuous variables were expressed as mean ± standard deviation and categorical variables as numbers (percentages). The normality of data was tested by Kolmogorov–Smirnov test (*K*–*S* test). Data were analyzed by Levene's test for equality of variances and compared by independent samples *T*-test or Chi-square test.

## Results

We collected 59 cases of distal femur fractures with a coronal fragment. The general information is shown in Table 1. 32% (*n* = 33/103) of AO/OTA type 33-C fractures were found to have a coronal fragment, including 4 cases were bicondylar coronal fractures, while 67% (31/46) of types 33-C3 had coronal fragments. No cases of 33-B3-1 or B3-3 fractures were found in our study. According to Letenneur's classification, Busch–Hoffa fractures (AO/OTA type 33-B3-2) were grouped into types I (*n* = 15), IIa (*n* = 6), IIb (*n* = 2), and III (*n* = 5). The angles of the fractures are also shown in Table 1. In the type 33-B3 group, the angle *α* at the lateral condyle was larger than at the medial condyle (35.8 ± 10.9° vs. 21.9 ± 12.5°, *p* = 0.004); but the opposite was found in type 33-C3 group (9.3 ± 13.3° vs. 28.8 ± 13.6°, *p* = 0.001). The angles for types 33-B3 and 33-C3 in the medial condyle were comparable. However, at the lateral condyle, both angles *α* and *β* were smaller in type 33-C3 than in type 33-B3 (angle *α*: 9.3 ± 13.3° vs. 35.8 ± 10.9°, *p* < 0.001; angle *β*: 14.8 ± 14.8° vs. 26.8 ± 8.6°, *p* = 0.002).

**Table 1:** General information of patients with coronal fractures

	Type 33-B3	Type 33-C3	*p*
Number^a^	28 (100)	31 (100)	
Sex^a^			0.08
Male	19 (68)	14 (45)	
Female	9 (32)	17 (55)	
Ages^b^	45 (14)	54 (16)	0.019
Injuried side^a^			–
Left knee	17 (61)	10 (32)	
Right knee	11 (39)	21 (68)	
Injured condyle^a^			–
Lateral condyle	14 (22)	22 (71)	
Medial condyle	14 (22)	5 (16)	
Bicondyle	0	4 (13)	
Angle *α*			
Lateral condyle	35.8 ± 10.9°	9.3 ± 13.3°	< 0.001
Medial condyle	21.9 ± 12.5°	28.8 ± 13.6°	0.229
*p*	0.004	0.001	–
Angle *β*			
Lateral condyle	26.8 ± 8.6°	14.8 ± 14.8°	0.002
Medial condyle	24.6 ± 12.6°	18.8 ± 13.1°	0.305
*p*	0.581	0.479	–

^a^Categorical variables are shown as the number (percentage)

^b^Continuous variables are shown as the mean (standard deviation)

### Three-dimensional maps

**Fig. 2:**
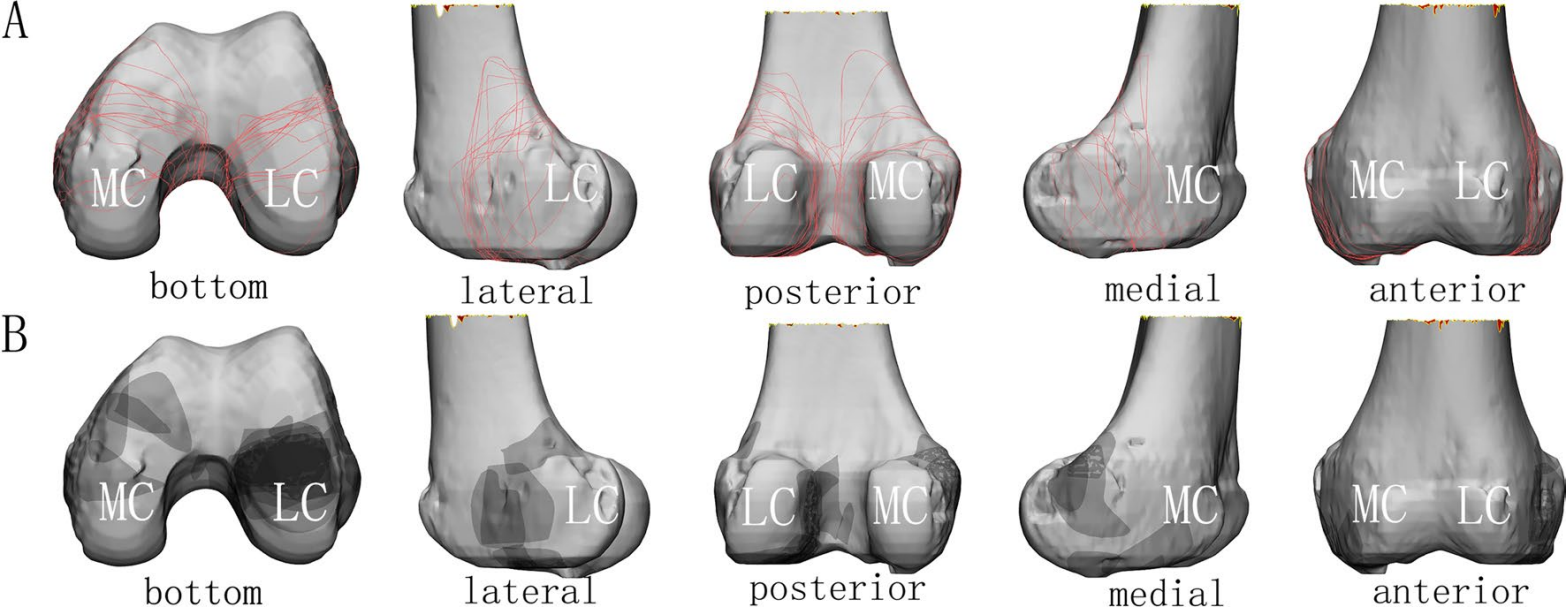
Map of type 33-B3 fracture. **A** Fracture lines; **B** comminution zone. MC, Medial condyle; LC, lateral condyle

The type 33-B3 group (*n* = 28) (Fig. 2) contained 14 lateral and 14 medial fractures. On bottom views, the lateral fractures were concentrated at the middle of the condyle. On lateral views, the fractures extended to the posterosuperior epicondylar area. On posterior views, the fractures converged toward the anterolateral intercondylar fossa. The medial condyle fracture planes were similar but more dispersed. The concentration of the comminution area (Fig. 2B) is clearly seen in the bottom view. Since the number of the comminuted medial fractures was too small, the comminution area could not be quantified.

**Fig. 3:**
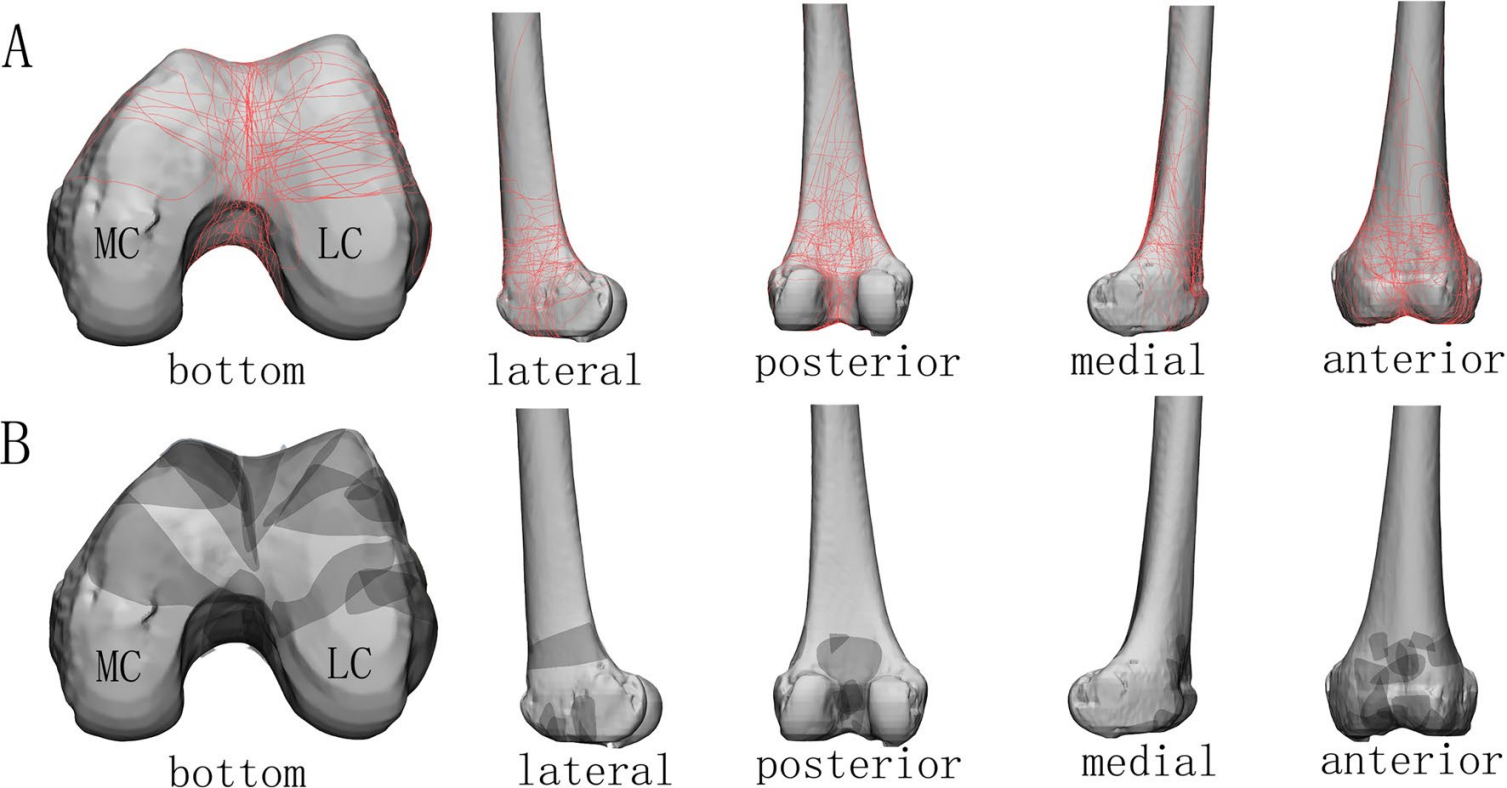
Map of type 33-C3 fractures with the most common coronal fragment. **A** Fracture lines; **B** comminution zone. MC, medial condyle; LC, lateral condyle

The type 33-C3 group (*n* = 31) (Fig. 3) consisted of lateral (*n* = 22), medial (*n* = 5) and bicondylar (*n* = 4) coronal fractures. On bottom and lateral views, the coronal fractures at the lateral condyle were concentrated at the junction of the anterior and middle thirds. On posterior views, the fractures converged toward the intercondylar fossa. The fracture lines at the medial condyle were similar but more dispersed. The comminution or collapse of the articular surface was rarely observed and occurred at the patellofemoral joint, the intercondylar fossa, or the anterior condyle.

**Fig. 4:**
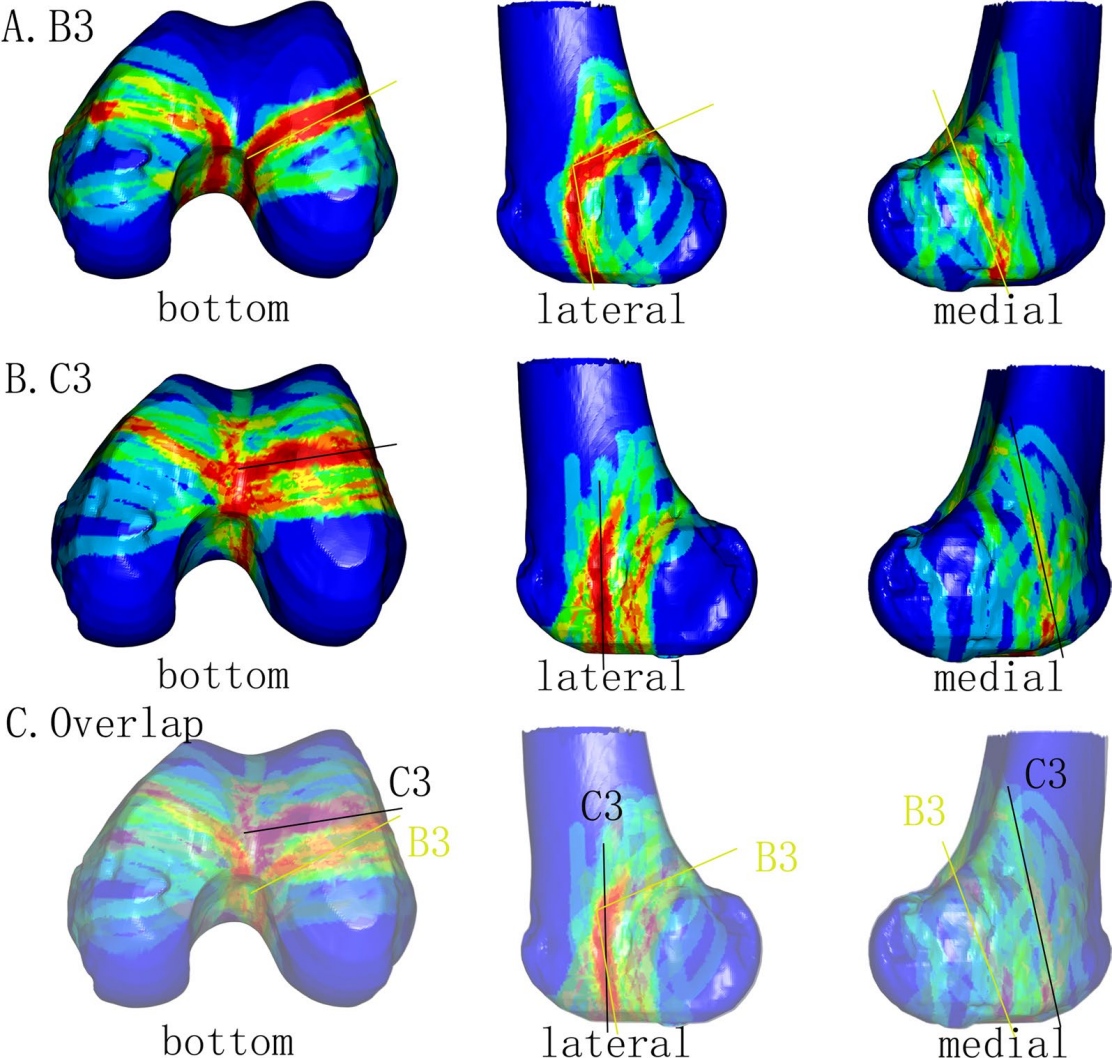
Heat maps of type B3 and C3 fractures. **A** Maps of type B3. The yellow lines indicate the hot zone on the lateral condyle. **B** Maps of type C3. The black lines indicate the hot zone (high-density fracture zone). **C** Overlap of the maps

The heat maps of the coronal fractures in type B3 and C3 fractures are shown in Fig. 4A, B (fracture lines are deleted if they are not related to the coronal plane fracture). Figure 4C shows the overlap of the heat maps. The high-density fracture area for type C3 fractures was anterior to that of type B3 fractures in the bottom view for both condyles, while the high-density fracture area of both fractures overlapped at the bottom of the lateral view. The coronal fracture lines of type C3 were interrupted by the metaphyseal fracture lines, but the fracture lines of type B3 were continuous and converged toward the intercondylar fossa. Accordingly, the high-density area for both types of fractures were different at the epicondyle region in the lateral view. The hot zones on the medial condyle for both fractures were dispersed.

## Discussion

In the present study, we generated 3D maps of the distal femur fractures with coronal fragments and measured the angles of the coronal fractures. Coronal fractures occurred in approximately 32% of the distal femur fractures, involving 67% of type 33-C3 fractures. The coronal fractures were more likely to occur at the lateral condyle [5], which may result from the valgus of the distal femur. It is widely acknowledged that compared with plain radiographs, CT scan imaging makes it easier to identify coronal fragments [5, 14, 15].

Overwhelming evidence suggests that the incidence of coronal fractures is relatively high. Nork et al. identified coronal fragments in 38.1% of supracondylar–intercondylar fractures, and open fractures were more likely to have coronal fragments than closed fractures [5]. Richards et al. found that 53% of supracondylar–intercondylar fractures had coronal fractures [16]. Li et al. [4] reported that coronal fractures were present in 41.1% of articular fractures, being involved in 36.8% of AO/OTA type C and comprising 48.1% of type B fractures. In the present study, 67% of type 33-C3 fractures were diagnosed with coronal fractures. Biplanar plain radiographs were diagnostic only for 69% of coronal plane fragments [5], emphasizing that a preoperative CT scan is mandatory to avoid missed diagnoses.

A three-dimensional fracture map can demonstrate the characteristic of fracture morphology and has been used to study fractures in distal radius [17], intertrochanter, distal femur [4], patella [18], tibial plateau [19], and ankle [20], providing more details and the overall characteristics of fractures compared to plain radiography or two-dimensional CT images. Based on the 3D fracture map, the fracture heat map also displays the fracture incidence of each part. Although the fracture heatmap highlights frequently fractured sites, some rare fracture types can be ignored. It should be borne in mind that the 3D fracture map also has its shortcomings: if too many types or numbers of fractures are included, the characteristics of each type of fracture cannot be clearly displayed. Therefore, it is necessary to effectively classify fractures when drawing the map.

Richards et al. divided the intercondylar coronal fractures into four regions: anteromedial, posteromedial, anterolateral, and posterolateral [16]. In their study, the comminuted area of the articular surface was in the weight-bearing area, similar to comminution in the Busch–Hoffa fracture [12, 16]. However, in our 3D maps, the comminution zone and the fractures in type 33-C3 coronal fractures were anterior to that in the Busch–Hoffa fracture. The reconstruction of type 33-C3 fractures showed that most coronal fracture lines ended at the metaphyseal and patellofemoral articular fractures, suggesting that both metaphyseal and patellofemoral fractures occurred prior to fracture of the posterior condyle and that the isolated Busch–Hoffa fracture occurred with both regions intact. This discrepancy may be caused by the different injury mechanisms and resulted in two kinds of comminution and fractures.

The fracture lines and the comminuted area in type 33-B3 in our study were similar to those reported by Xie et al. [13], who found that the angles between the Busch–Hoffa fracture and the posterior condyle axis were 34.4° (range, − 8.4° to 52.7°) at the lateral condyle and 29.0° (range, − 19.4° to 59.4°) at the medial condyle; the fracture angles with the distal femoral shaft were 23.1° (range, − 8.2° to 68.2°) at the lateral condyle and 19.3° (range, − 10.8° to 58.6°) at the medial condyle. The lateral angles in this study were similar, but the medial angles differed, which may be caused by the dispersion of the distribution of the fractures lines in the medial condyle and the small number of the samples (*n* = 26 in Xie et al.'s study, *n* = 14 in the present study).

A mechanical study by Jarit et al. demonstrated that posterior–anterior screws were more stable than anterior–posterior screws for AO/OTA type 33-B3 [9]. Their samples were obtained by osteotomy along the posterior cortex of the femur. However, in the present study, Busch–Hoffa fractures were anterior to the posterior cortex and extended in an anteroinferior direction, consistent with Xie et al.'s findings. The fractures of Jarit's samples were posterior to the concentrated area on the fracture map. Currently, there is no standard osteotomy or fracture model for distal femur biomechanical studies. Importantly, fracture mapping enables visualization of the fracture characteristics and can be used as a reference for making fracture models. Li et al. [4] suggested that fracture models should be made according to their morphology results.

Our study has several limitations and shortcomings. Injury mechanism and patient outcomes were not analyzed. Patients were excluded from our research if they were diagnosed by plain radiography or CT scan imaging data was unavailable. Finally, this was a single-center study, which compromises the robustness of our findings.

## Conclusion

We found that 32% of type 33-C fractures presented coronal fractures, and most were type C3 fractures. The morphology of the coronal fractures in the C3 group was different from those in the 33-B3 group; the coronal fractures in type 33-C3 were parallel to the coronal plane and anterior to the intercondylar fossa, with comminution on the anterior condyle.

## Data Availability

The morphology of the coronal fractures in the femoral supracondylar and intercondylar fractures © 2022 by Jun Lin Zhou is licensed under Attribution-NonCommercial-Share Alike 4.0 International. To view a copy of this license, visit http://creativecommons.org/licenses/by-nc-sa/4.0/. The dataset supporting the conclusions of this article is available in the open science framework, Identifier: https://doi.org/10.17605/OSF.IO/HKG3X, https://osf.io/hkg3x/?view_only=c5e36d9b2a7c4e6796017d87fb1ceedd. CT images are not provided to respect patient privacy, but are available from the corresponding author on reasonable request.

## Abbreviations

CT: Computed tomography
AO/OTA: Arbeitsgemeinschaft für Osteosynthesefragen/Orthopaedic Trauma Association
3D: Three-dimensional
